# Symmetrical Drug-Related Intertriginous and Flexural Exanthema (SDRIFE): A Case Report

**DOI:** 10.7759/cureus.110058

**Published:** 2026-06-01

**Authors:** Ruba J Almahmudi, Hamza A Alzahrani, Ascia K Alabbasi, Ghaidaa Almatrafi, Hadeel A Alharbi, Khalid Al Hawsawi

**Affiliations:** 1 Medicine, Umm Al-Qura University, Makkah, SAU; 2 Dermatology, King Abdulaziz Hospital, Makkah, SAU

**Keywords:** dermatologic drug reaction, erythematous patches drug eruption, fluconazole, prednisolone treatment, symmetrical drug-related intertriginous and flexural exanthema

## Abstract

Symmetrical drug-related intertriginous and flexural exanthema (SDRIFE) is an interesting phenomenon of drug response characterized by symmetrical, exuberant skin reactions often seen in flexural and intertriginous areas. This report describes a 35-year-old gentleman with no relevant clinical history who developed SDRIFE subsequent to the use of oral fluconazole. His skin examination showed non-scaly, erythematous to violaceous patches within the genital, antecubital, axillary, neck, and inframammary folds. The diagnosis was made based on the clinical picture. Discontinuation of fluconazole and a tapering course of oral prednisolone led to complete resolution of symptoms. This case underlines the importance of maintaining a low threshold of suspicion for rare drug reactions such as SDRIFE, even with medications that are not commonly viewed as requiring dermatological follow-up, so that the condition can be directly managed and hopefully prevented in the future.

## Introduction

Drug-induced intertrigo, also known as symmetrical drug-related intertriginous and flexural exanthema (SDRIFE), is classified as a drug eruption specific to a drug that causes symmetrical erythematous patches that mostly appear in the flexural areas of the body upon systemic administration of a drug [1, 2]. It usually involves the anogenital region and at least one other flexural region, such as the axilla, neck, antecubital fossa, or inframammary area [3, 4]. Some authors use the term "baboon syndrome" when the buttocks are involved. Rashes in SDRIFE are typically bright red and well delineated, flat or slightly raised, and do not accompany scaling. These usually occur within hours to a few days after intake of the offending drug. Unlike other drug eruptions, SDRIFE affects the flexural areas with no related systemic symptoms such as fever or internal organ involvement [5]. In the case of antimicrobials and antifungals, drugs reported to trigger SDRIFE include clindamycin, fluconazole, other beta-lactams and macrolides, and terbinafine [3, 4]. The uncertainty surrounding the issue is attributed to locally stimulated inflammation mediated by a Type IV hypersensitivity T-cell response [5, 6]. Several medications, as well as vaccines, have been associated with SDRIFE [1, 5]. The diagnostic criteria include (a) taking a systemic drug that is not a chemotherapeutic agent, (b) prominent redness around the anogenital area, (c) redness in at least one more intertriginous or flexural area, (d) bilateral (symmetrical) involvement, and (e) no systemic symptoms, such as fever or internal organ involvement [7-9]. SDRIFE must be differentiated from other drug eruptions such as morbilliform drug eruption, fixed drug eruption, acute generalized exanthematous pustulosis (AGEP), and classic baboon syndrome. Morbilliform eruption is characterized by a diffuse maculopapular rash, typically present on the trunk and limbs. Fixed drug eruption recurs at the same site after re-exposure and may leave residual hyperpigmentation. Sterile pustules characterize AGEP, which is often associated with fever [10]. In contrast, SDRIFE is characterized by symmetrical erythema limited mainly to flexural areas, with no systemic manifestations [1].

## Case presentation

**Figure 1:**
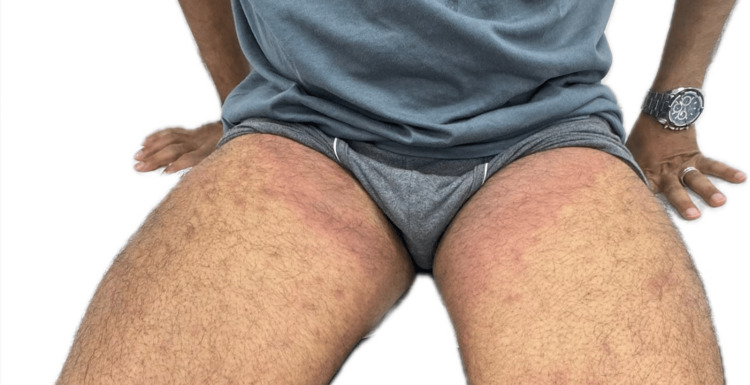
Similar lesions involving the inguinal and genital areas, as well as the thighs, predominantly affecting the flexural regions

**Figure 2:**
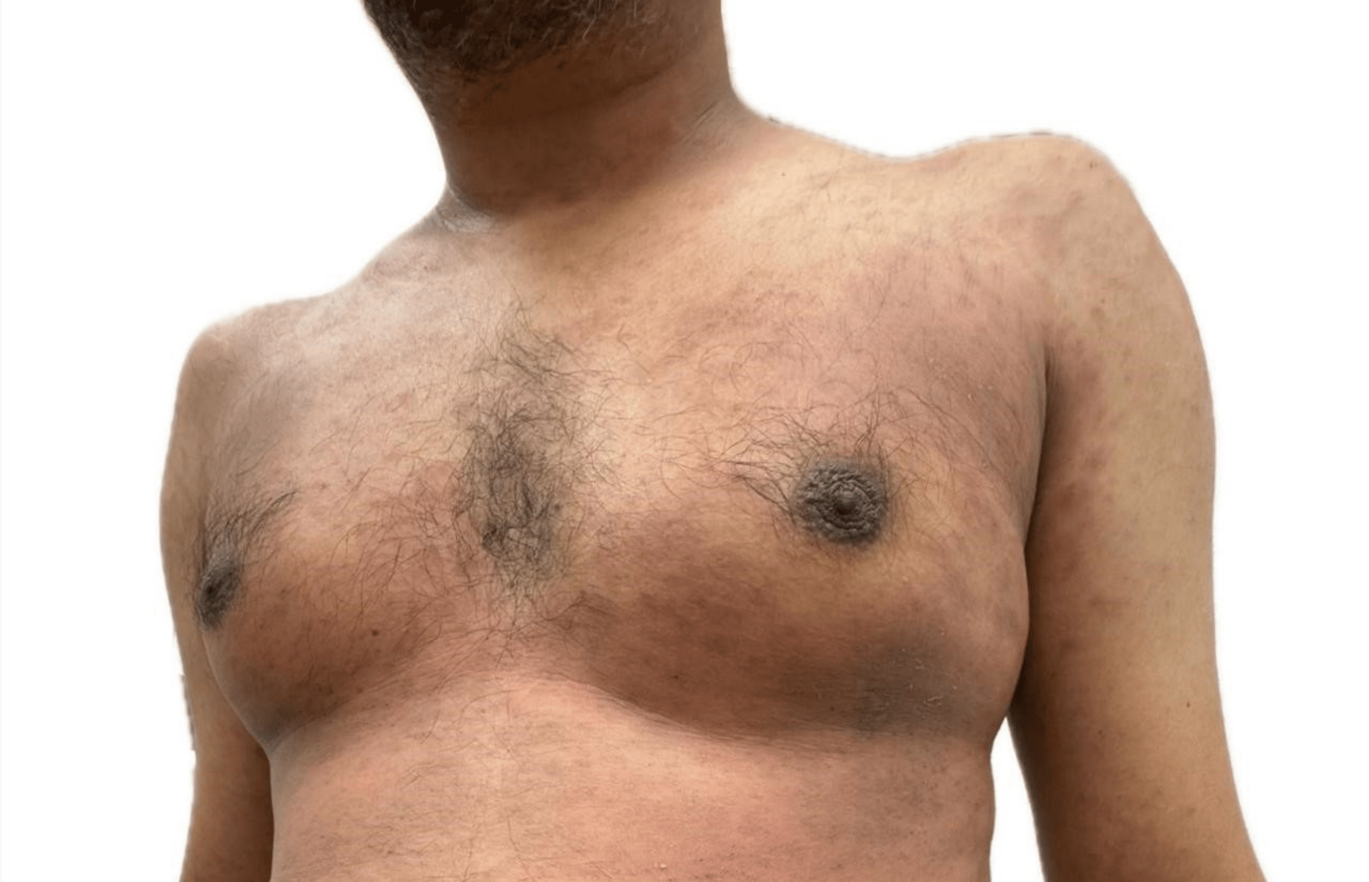
Diffuse, non-scaly erythematous patches involving the neck, inframammary, and axillary areas

A 35-year-old healthy man presented with very itchy and persistent skin lesions. He had received oral fluconazole 150 mg once weekly prophylactically. Two weeks earlier, his wife had seen a dermatologist, was diagnosed with tinea cruris, and was prescribed fluconazole. Both husband and wife had been prescribed fluconazole by the dermatologist to avoid the transfer of the fungus between them. However, the patient did not have any clinical or mycological evidence of a fungal infection. There was no history of fever, joint pain, or myalgia. The systems review was unremarkable, with no significant medical or family history. Cutaneous examination showed non-scaly, erythematous to violaceous lesions that were mostly distributed over the flexural and intertriginous areas, such as the genital, thigh, axilla, inframammary, antecubital fossae, and neck regions (Figures 1, 2). Other areas beyond the flexural regions were also involved, indicating a more generalized distribution. Hair, nails, and mucous membranes were normal. 

Laboratory investigations were performed to exclude systemic involvement (Tables 1, 2).

**Table 1:** Complete blood count (CBC) MONO: monocytes; EOS: eosinophils.

Test	Result	Unit	Normal Range
MONO%	10.6 ↑	%	2-10
EOS%	0.8 ↓	%	1-6

**Table 2:** Biochemistry results (liver, kidney, and electrolytes)

Test	Result	Unit	Reference Range
Creatinine	114	µmol/L	65.4-110.5
Chloride	108	mmol/L	98-107

Although the majority of laboratory values were normal, there was a mild increase in monocytes and slight increases in creatinine and chloride, along with a mild decrease in eosinophils, none of which were clinically significant. Based on the above findings, the patient was diagnosed with fluconazole-induced SDRIFE. This was supported by the lack of systemic symptoms, temporal relationship to drug exposure, and characteristically symmetrical distribution over the intertriginous and flexural sites, consistent with the diagnostic criteria of SDRIFE. Oral fluconazole was immediately stopped. Oral prednisolone 30 mg daily was initiated for two weeks. The patient was advised to refrain from future use of fluconazole, and the rash completely subsided after two weeks.

## Discussion

SDRIFE was first described in 1984, and since then, only around 100 cases have been reported in the literature [7]. It is a benign skin condition that typically affects the intertriginous, inguinal, and gluteal folds following systemic drug exposure. The most commonly reported drugs include amoxicillin, beta-lactams, clindamycin, macrolides, azoles, and contrast agents [11]. Other reported medications include antibiotics, antivirals, allopurinol, acetaminophen, hydrochlorothiazide (HCTZ)/telmisartan, IVIg, nonsteroidal anti-inflammatory drugs (NSAIDs), proton pump inhibitors, and many others. Chemotherapeutic agents usually cause toxic erythema of chemotherapy, which has a similar clinical presentation and usually occurs two to four weeks after exposure to the offending chemotherapeutic agent.

In SDRIFE, patients usually develop a rash within hours to two days after systemic administration of the offending medication, especially with re-exposure [11]. The exact mechanism of SDRIFE remains unclear, although it is believed to be caused by a Type IV hypersensitivity immune response [12].

Prophylactic therapy with systemic or topical antifungal agents is not recommended to prevent the emergence of fungal infection. No such measures should be recommended unless substantiated by literature because of the risk of severe drug side effects. The patient received a second dose of fluconazole two days later, after which he developed a skin rash. The patient experienced an all-over rash that was determined to be a drug-induced hypersensitivity reaction. As a triazole, fluconazole is classified as an antifungal agent. The mechanism of action of fluconazole involves blocking 14-alpha-demethylation of the fungal cellular enzyme, thereby altering the composition of the fungal cell membrane by inhibiting ergosterol production. The hypersensitivity reaction described here represents an example of a delayed reaction.

The latency period between the culprit drug and onset of the skin rash was not longer than classically described for the following reasons: the dose of fluconazole was administered once weekly, the patient had no prior fluconazole exposure, and the first dose of fluconazole elicited no reaction, whereas a drug rash developed shortly after the second dose. This is typical of SDRIFE.

Clinically, SDRIFE is characterized by sharply demarcated, V-shaped erythema in the gluteal and perianal areas, as well as in the axilla, antecubital fossa, and neck, with a symmetrical distribution [11, 12]. Our patient presented with all diagnostic criteria of SDRIFE, including non-scaly erythematous patches localized to the genital area, body flexures, and intertriginous areas. The culprit drug was fluconazole, which is among the commonly reported causes. He had no systemic manifestations, either clinically or laboratory-wise. Treatment includes discontinuation of the suspected drug along with symptomatic management. Prednisolone was used in our patient as symptomatic treatment.

## Conclusions

This case highlights the clinical significance of SDRIFE, a rare but recognizable type of drug eruption. Diagnosis is clinical and is supported by a clear temporal relationship with drug administration and exclusion of alternative causes, especially toxic erythema of chemotherapy. This case reinforces the importance of clinician awareness of rare cutaneous adverse drug reactions to ensure timely diagnosis, appropriate treatment, and prevention of future incidents related to drug exposure.
